# The impact of obesity severity on oxidative stress, exercise-related biomarkers, and physical activity in type 2 diabetes: a study including subjective sleep assessment

**DOI:** 10.1590/1806-9282.20252106

**Published:** 2026-06-29

**Authors:** Emine Cihan, Gulsum Abusoglu, Melek Altunkaya, Cansu Sahbaz Pirincci, Hasan Gerçek, Ülkü Saygılı Duzova, Murat Akay, Ahmet Gorgel, Sedat Abusoglu, Suleyman Baldane

**Affiliations:** 1 Selcuk University, Vocational School of Health Sciences – Konya, Türkiye.; 2 University of Health Sciences, Gulhane Faculty of Physiotherapy and Rehabilitation – Ankara, Türkiye.; 3 Acıbadem University, Faculty of Health Sciences, Department of Physical Therapy and Rehabilitation – Istanbul, Türkiye.; 4 Selçuk University, Faculty of Nursing, Department of Internal Medicine Nursing – Konya, Türkiye.; 5 Selcuk University, Faculty of Medicine, Department of Internal Medicine, Division of Endocrinology and Metabolism – Konya, Türkiye.; 6 Selcuk University, Faculty of Science, Department of Biochemistry – Konya, Türkiye.

**Keywords:** Type 2 diabetes mellitus, Obesity, Oxidative stress, Physical activity, Sleep quality

## Abstract

**OBJECTIVE:**

Type 2 diabetes mellitus is a chronic metabolic disorder characterized by insulin resistance and β-cell dysfunction, often coexisting with obesity. Obesity increases adipose tissue inflammation and oxidative stress, which may impair mitochondrial function and muscle metabolism. The aim of this study was to investigate the effects of obesity severity on oxidative stress biomarkers, exercise-related biomarkers, physical activity level, and sleep quality in individuals with type 2 diabetes mellitus.

**METHODS:**

This prospective observational study included 80 individuals aged 20–80 diagnosed with type 2 diabetes mellitus. Participants’ body mass index, physical activity assessed by the International Physical Activity Questionnaire-Short Form, and sleep quality assessed by the Pittsburgh Sleep Quality Index were recorded. Serum samples were analyzed for oxidative stress biomarkers (total antioxidant status, total oxidant status, malondialdehyde, and superoxide dismutase) and exercise-related biomarkers (irisin, Fibronectin Type III Domain-Containing Protein 5, Adenosine Monophosphate-Activated Protein Kinase, and leptin) using enzyme-linked immunosorbent assay methods.

**RESULTS:**

There were significant differences in irisin, Fibronectin Type III Domain-Containing Protein 5, Adenosine Monophosphate-Activated Protein Kinase, leptin, malondialdehyde, superoxide dismutase, total antioxidant status, and total oxidant status values according to body mass index levels (p<0.001). Overweight participants had higher irisin, Adenosine Monophosphate-Activated Protein Kinase, and Fibronectin Type III Domain-Containing Protein 5 levels, while leptin, malondialdehyde, and total oxidant status levels increased with obesity severity. Physical activity levels decreased as body mass index increased (p=0.016). No significant differences were observed in self-reported sleep quality parameters assessed by the Pittsburgh Sleep Quality Index across body mass index groups (p>0.05).

**CONCLUSION:**

Increasing obesity severity in individuals with type 2 diabetes mellitus is associated with elevated oxidative stress and decreased antioxidant defense, along with lower levels of exercise-related metabolic biomarkers. These findings highlight the importance of promoting regular physical activity and interventions targeting the irisin/Adenosine Monophosphate-Activated Protein Kinase pathway to reduce oxidative stress and improve metabolic health in obese diabetic individuals.

## INTRODUCTION

Type 2 diabetes (T2DM) is a chronic disease defined as a global public health problem where insulin resistance and progressive β-cell dysfunction are observed intensely^1^. Obesity, one of the risk factors, is defined as a chronic complex metabolic disorder characterized by excessive fat accumulation in the body, which also has destructive effects on the body^1,2^. Adipose tissue, which increases with obesity, begins to function as an endocrine organ over time; that is, it secretes biomarkers that increase inflammation in the body, causing the body to be exposed to chronic inflammation^3^.

Oxidative and exercise biomarkers provide information regarding body dynamics in areas such as mitochondrial function, muscle performance, body inflammation, and metabolic health^4^. Due to the development of hyperglycemia in T2DM, increased excessive glucose entry into the cell, and the impairment of mitochondrial dysfunction, abnormalities are observed in oxidative stress mechanisms, and changes may occur in oxidative stress biomarker levels^3,5^.

Physical activity supports mitochondrial biogenesis and oxidative muscle fiber development in muscle tissue, while also increasing Adenosine Monophosphate-Activated Protein Kinase [AMPK] activation by strengthening antioxidant defense systems^6^. Regular and moderate physical activity ensures the balancing and improvement of oxidative stress biomarkers and exercise-related biomarkers^5,7^. Poor sleep can also lead to the development of factors that negatively affect the body's repair and adaptation, such as changes in cortisol levels, impairment of mitochondrial function, decreased growth hormone levels, and slowing of protein synthesis^8,9^. This study aimed to compare oxidative stress biomarkers, exercise-related biomarkers, physical activity, and sleep quality according to obesity levels in patients with T2DM.

## METHODS

### Study design

This study is a prospective observational, single-center research. The study was carried out after receiving approval (no. 2024/193) from the Selçuk University Ethics Committee. The research was conducted in adherence to the principles stated in the Declaration of Helsinki.

The selection of patients included in the study was performed using the random sampling method. Based on the data obtained from the study by Nolan et al.^10^ it was calculated that at least 64 individuals should be included with an effect size of 0.43, 80% power, and a 0.05 Type I error. Considering potential data loss, 80 individuals (20%) were included in the study.

The patients included in the study consisted of individuals aged 20–80 years, diagnosed with T2DM for at least 6 months, and capable of answering the questionnaires cognitively and physically. A diagnosis of T1DM, history of infectious disease, malignancy, liver or kidney disease, muscle/joint pathologies, severe neurological or psychiatric disorders, and pregnancy or lactation periods were exclusion criteria.

### Collection, storage, and analysis of biomarkers

In the study, routine blood tests of 80 patients were performed, and samples were selected from blood specimens that had results and were sent to the biological waste. No extra blood samples were taken from the patient. Samples were stored at −80°C until the study day. On the study day, samples removed from −80°C were thawed at room temperature and homogenized. For biomarker analysis, standards were prepared according to the protocol and added to the 96-well plate together with the samples. Buffer solution was added to each well and incubated for 90 min at room temperature. Necessary washings were performed after each stage, and finally, chromogenic agents were added, and spectrophotometric readings were performed. Although the specified timing and additions may vary according to the parameter, the general procedure is as stated.

All reagents of the enzyme-linked immunosorbent assay (ELISA) kits for oxidative stress markers, total antioxidant status (TAS), total oxidant status (TOS), malondialdehyde (MDA), and superoxide dismutase (SOD) were brought to room temperature. Each sample was run in duplicate for both TAS and TOS. All reagents of the ELISA kits for exercise-related markers, irisin, AMPK, Fibronectin Type III Domain-Containing Protein 5 (FNDC5), and leptin were brought to room temperature. Each sample was run in duplicate for both irisin and FNDC5^11^.

### International Physical Activity Questionnaire-Short Form

It evaluates physical activity levels within the last 7 days as vigorous, moderate, walking, and sitting. When calculating the total score, the duration of the activity is multiplied by the frequency of the activity to obtain a score in metabolic syndrome. As the total score increases, the individual's physical activity level is interpreted as high^12^.

### Pittsburgh Sleep Quality Index

The PSQI is a widely used and validated subjective self-report measure that evaluates sleep quality and sleep disturbances over the past month. The scale consists of seven components. Each component is evaluated over 0–3 points. A total score greater than five indicates "poor sleep quality"^13^.

### Statistics

International Business Machines Statistical Package for the Social Sciences Statistics v. 29.0 was used for statistical analyses. The homogeneity of variances, a prerequisite for parametric tests, was checked with the "Levene" test. The assumption of normality was examined with the "Shapiro-Wilk" test. Since the data didn't follow a normal distribution, the Kruskal-Wallis test was used for comparing three or more groups, and the Mann-Whitney U test was used for comparing paired groups. Spearman correlation analysis was performed to examine the associations between physical activity, body mass index (BMI), sleep quality, and selected metabolic and oxidative stress biomarkers. The significance level was accepted as p<0.05.

## RESULTS

The study included 80 individuals aged 20–80 with T2DM. The majority of the sample consisted of middle-aged and older adults. The socio-demographic information of the participants is provided in Table 1. The participants’ average IPAQ values were 747.52±672.74, while 38 out of 42 (52.5%) were physically inactive. The average PSQI values of the participants were 6.38±2.44, while 45 (56.2%) had poor sleep quality (Table 2).

**Table 1:** Participants’ socio-demographic characteristics.

		X±SD/n (%)
Age		56.81±10.21
BMI		35.48±6.12
Gender		
	Male	36 (45.0%)
	Female	44 (55.0%)
Marital status		
	Married	76 (95.0%)
	Single	4 (5.0%)
Chronic disease		
	Yes	48 (60.0%)
	No	32 (40.0%)
Educational status		
	Elementary	49 (61.3%)
	Middle	3 (3.7%)
	High	16 (20.0%)
	University	12 (15.0%)
Smoking		
	Yes	16 (20.0%)
	No	64 (80.0%)

X: mean; SD: standard deviation; n: number; %: percentage; BMI: body mass index.

**Table 2:** Participants’ physical activity levels and sleep quality.

		X±SD/n (%)
Physical activity		
	IPAQ	747.52±672.74
	Inactive	42 (52.5%)
	Minimally active	36 (45.0%)
	Very active	2 (2.5%)
Sleep quality		
	Duration	0.45±0.83
	Sleep disturbance	1.18±0.38
	Sleep duration	1.16±0.77
	Insomnia-related functional impairment	1.15±0.84
	Sleep efficiency	1.31±0.77
	Overall sleep quality	1.05±0.65
	Sleep medication	0.08±0.41
	Total	6.38±2.44
	Good sleep quality	35 (43.8%)
	Poor sleep quality	45 (56.2%)

X: mean; SD: standard deviation; n: number; %: percentage; IPAQ: International physical activity questionnaire.

Significant differences were observed in Irisin, AMPK, Leptin, FNDC5, MDA, SOD, TAS, and TOS levels across BMI groups (p<0.001). The overweight group showed higher Irisin, AMPK, FNDC5, and SOD levels than all obesity grades (p<0.001), while the obese groups displayed similar values. Leptin levels were comparable in Grade II and Grade III obesity (p=0.583) and higher than in the Grade I obesity and overweight groups (p<0.001). MDA levels were similarly elevated in Grade II and Grade III obesity (p=0.779). The TAS value was higher in the overweight group compared to the Grade I (p<0.001), Grade II (p=0.002), and Grade III (p<0.001) groups. The TOS value was lower in the overweight group compared to the Grade I, Grade II, and Grade III groups (p<0.001) (Table 3).

**Table 3:** Comparison of participants’ biomarkers according to their body mass index levels.

		Overweight (n=20) X±SD	Grade I Obesity (n=20) X±SD	Grade II Obesity (n=20) X±SD	Grade III Obesity (n=20) X±SD	H	p
Biomarkers							
	İrisin	14.69±10.27	5.68±2.18	5.30±3.29	5.24±2.13	30.773	**<0.001**
	AMPK	26.43±25.90	12.31±4.07	11.72±3.60	6.89±4.67	35.247	**<0.001**
	Leptin	0.64±0.69	0.85±0.72	1.64±0.43	2.08±1.07	29.437	**<0.001**
	FND5	86.60±56.90	30.51±15.48	24.37±12.82	11.63±5.04	43.309	**<0.001**
	MDA	8.87±16.16	6.51±4.23	15.22±12.86	19.76±20.65	23.746	**<0.001**
	SOD	73.69±34.38	53.51±14.13	31.93±8.06	25.96±16.85	44.840	**<0.001**
	TAS	4.52±0.96	3.48±0.61	3.98±1.12	3.15±1.36	19.324	**<0.001**
	TOS	2.12±0.76	3.92±2.27	3.08±0.64	3.93±0.84	32.168	**<0.001**

X: mean; SD: standard deviation; n: number; H: Kruskal-Wallis; MDA: malondialdehyde; SOD: superoxide dismutase; TAS: total antioxidant status; TOS: total oxidant status; AMPK: Adenosine Monophosphate-Activated Protein Kinase; pairwise comparisons were performed using Mann-Whitney U test; p<0.05. Bold formatting indicates statistically significant differences (p<0.05).

Sleep subparameters were similar across participants based on BMI levels. Overall sleep quality (p=0.974), sleep medication use (p=0.795), and total PSQI scores (p=0.467) were similar (Table 4).

**Table 4:** Comparison of participants’ sleep quality and physical activity levels according to their body mass index levels.

		Overweight (n=20) X±SD	Grade I Obesity (n=20) X±SD	Grade II Obesity (n=20) X±SD	Grade III Obesity (n=20) X±SD	H	p
PSQI							
	Duration	0.80±0.95	0.40±40.88	0.25±0.55	0.35±0.81	7.691	0.053
	Sleep disturbance	1.10±0.31	1.10±0.32	1.20±0.41	1.30±0.47	3.762	0.288
	Sleep duration	1.25±0.97	0.95±0.69	1.30±0.66	1.15±0.75	2.101	0.552
	Insomnia-related functional impairment	1.35±0.99	1.15±0.88	1.05±0.69	1.05±0.83	1.225	0.747
	Sleep efficiency	1.55±0.83	1.25±0.85	1.30±0.73	1.15±0.67	3.553	0.314
	Overall sleep quality	1.15±0.93	1.00±0.46	1.00±0.56	1.05±0.60	0.221	0.974
	Sleep medication	0.05±0.22	0.15±0.67	0.10±0.45	0.01±0.01	1.027	0.795
	Total	7.25±2.86	6.00±2.73	6.20±2.12	6.05±1.85	2.549	0.467
	IPAQ	1,012.35±602.47	464.70±416.74	915.43±896.58	597.60±574.38	10.302	**0.016**

X: mean; SD: standard deviation; n: number; PSQI: Pittsburg Sleep Quality Index; IPAQ: International Physical Activity Questionnaire; H: Kruskal-Wallis; p<0.05. Bold formatting indicates statistically significant differences (p<0.05).

There was a difference in physical activity levels according to BMI levels (p=0.016). Overweight individuals had higher physical activity levels than participants with Grade I (p=0.005) and Grade III obesity (p=0.023). Similarly, participants with Grade II obesity had higher physical activity levels than participants with Grade I obesity (p=0.043) (Table 4).

Participants’ BMI and TOS values showed a negative linear correlation (p=0.001, r=-0.351). There was no statistically significant correlation between the IPAQ with Irisin (p=0.643, r=-0.053) and PSQI with Leptin (p=0.335, r=-0.109) values of the participants (Table 5).

**Table 5:** The relationship between participants’ International Physical Activity Questionnaire, Irisin, body mass index, total oxidant status, Pittsburgh Sleep Quality Index, and leptin values.

	IPAQ—irisin	BMI—TOS	PSQI—leptin
p	0.643	**0.001**	0.335
r	-0.053	**-0.351**	-0.109

BMI: body mass index; PSQI: Pittsburg Sleep Quality Index; IPAQ: International Physical Activity Questionnaire; r: Spearman's Rho; TOS: total oxidant status; p<0.005. Bold formatting indicates statistically significant differences (p<0.05).

## DISCUSSION

In this study, the effects of the degree of obesity on oxidative stress, exercise-related biomarkers, physical activity, and sleep quality were examined in individuals with T2DM. The sample consisted of middle-aged and older individuals, predominantly women, married, with low educational attainment and high BMI values; these characteristics are consistent with the known sociodemographic risk profile for T2DM^1,14^.

No differences were observed between BMI-based groups in terms of sleep quality and physical activity levels, nor in sleep quality subcomponents (sleep duration, sleep disturbance, sleep efficiency, overall sleep quality, daytime dysfunction due to insomnia, and use of sleep medication). Although this finding may suggest that increasing obesity severity does not lead to a marked differentiation in self-reported PSQI scores, it does not imply the absence of true sleep pathology as assessed by objective methods such as polysomnography. Highly prevalent conditions such as obstructive sleep apnea (OSA), which cannot be identified using PSQI, may have contributed to oxidative stress and metabolic alterations independently of perceived sleep quality. However, studies employing large samples and objective measurements have reported significant associations between impaired sleep quality, glycemic control, and obesity^15,16^. Several possible reasons may explain the lack of differences observed in our study. First, since all participants had a diagnosis of T2DM, diabetes-related complications and glycemic fluctuations may have caused prevalent sleep disturbances across all groups, potentially "masking" the additional effect attributable to obesity^15,17^. Second, the PSQI does not directly assess specific sleep disorders such as OSA or circadian rhythm disturbances; instead, it reflects subjective sleep experience, and the limited sample size within each group may have reduced the statistical power to detect potential differences between BMI categories^16,17^. Third, sleep disturbances in obesity and T2DM have been shown to involve multifactorial mechanisms, including circadian hormone dysregulation—particularly melatonin—elevated leptin levels and other adipokine alterations, inflammation, and oxidative stress^2,16-19^.

The role of melatonin is especially noteworthy. Melatonin is not only a regulator of circadian sleep rhythms but also a potent antioxidant and a modulator of insulin sensitivity and adipose tissue function. Its secretion is commonly disrupted in obesity and T2DM. Moreover, documented interactions exist between melatonin and sex steroid hormones, which may further influence metabolic health and body composition^20^. In a gynecological context, melatonin has been shown to have therapeutic potential in conditions such as polycystic ovary syndrome^21^ and to interact with thyroid function during menopause^22^. This complex pathophysiology may not be fully captured by PSQI alone. Therefore, the use of this subjective questionnaire represents a limitation of this study, as it reflects perceived sleep quality rather than objective sleep parameters.

Between-group differences in HbA1c levels, diabetes duration, and hypoglycemic treatments (particularly the AMPK activator metformin) represent potential confounding factors; the lack of balance in these variables should be considered a limitation when interpreting differences in the irisin–AMPK–oxidative stress axis^18,23^. In our study, higher irisin/FNDC5 levels in the overweight group compared with the obese group are consistent with the fact that irisin is primarily a skeletal muscle-derived myokine and is strongly associated with muscle mass and physical activity^7,24^. In moderate obesity, relatively preserved muscle mass and physical activity may allow more pronounced irisin-mediated AMPK activation and related metabolic adaptations, including increased glucose uptake, enhanced fatty acid oxidation, and adipose tissue browning^6,7,24^. In severe obesity, however, sarcopenic obesity, sedentary behavior, and mitochondrial dysfunction associated with longer diabetes duration may suppress irisin production and AMPK activity^6,24^. Indeed, the literature indicates that low irisin levels and reduced AMPK activity are associated with insulin resistance, poor glycemic control, and increased oxidative stress, and that irisin-based interventions improve glycemic regulation and redox balance via AMPK activation in experimental diabetes models^6,25^.

In our study, the progressive increase in MDA and TOS levels, along with decreases in SOD and TAS as obesity severity increased, appears consistent with impairment of the irisin–AMPK axis. Accordingly, our findings support an integrative hypothesis suggesting that, as obesity progresses in individuals with T2DM, the irisin/AMPK-mediated protective response becomes exhausted, and this depletion, combined with increased oxidative stress burden, exacerbates the metabolic profile,^6,7,24^.

Nevertheless, the lack of balance between groups regarding HbA1c levels, diabetes duration, and particularly metformin therapy—which is known to activate AMPK—represents an important limitation of our study, given the potential confounding effects of these variables on irisin/AMPK signaling and oxidative stress markers^6,23^. On the other hand, the parallel increase in leptin levels with increasing obesity severity observed in our study supports the direct relationship between this hormone and adipose tissue mass and is consistent with the literature linking leptin resistance, increased cardiometabolic risk, and inflammation in obese patients with T2DM^6^.

In conclusion, obesity severity worsens oxidative stress and metabolic hormone profiles in patients with T2DM; although subjective sleep quality appears similar across groups, it likely masks underlying objective sleep pathology. Considering that sleep disturbances in obesity and T2DM are associated with multiple mechanisms—including OSA, circadian hormone dysregulation (e.g., melatonin), neuropathy, glycemic variability, and depression^6,20,22^ future studies incorporating objective sleep assessments such as polysomnography or actigraphy, alongside evaluation of HbA1c levels, diabetes duration, detailed information on the use of specific medications such as metformin known to directly affect AMPK activation, and assessment of circadian markers such as melatonin, would provide a more comprehensive understanding of the obesity–sleep–metabolism interaction.

## Data Availability

The datasets generated and/or analyzed during the current study are available from the corresponding author upon reasonable request.
